# The Peacock study: feasibility of the dynamic characterisation of the paediatric hypothalamic-pituitary-adrenal function during and after cardiac surgery

**DOI:** 10.1186/s12872-020-01516-y

**Published:** 2020-05-25

**Authors:** Daniel Paul Fudulu, Gianni Davide Angelini, Fani Fanoula Papadopoulou, Jonathan Evans, Terrie Walker-Smith, Ido Kema, Martijn Van Fassen, Serban Stoica, Massimo Caputo, Stafford Lightman, Benjamin Gibbison

**Affiliations:** 1 Department of Cardiac Surgery, Bristol Heart Institute, Bristol, UK; 2 Henry Welcome Laboratories for Integrative Neuroscience and Endocrinology, University of Bristol, Bristol, UK; 3 Department of Congenital Heart Surgery, Bristol Royal Hospital for Children, Bristol, UK; 4 Clinical Trial and Evaluation Unit, University of Bristol, Bristol, UK; 5 Department of Laboratory Medicine, University of Groningen, Groningen, Netherlands; 6 Department of Cardiac Anaesthesia, Bristol Heart Institute, Bristol, UK

**Keywords:** Glucocorticoids, Hypothalamus-pituitary-adrenal axis, Children, Cardiopulmonary bypass, Paediatric heart surgery

## Abstract

**Background:**

Cortisol is the main stress hormone mobilised during surgery to establish homeostasis. Our current understanding of the hypothalamic-pituitary-adrenal axis physiology in children undergoing cardiopulmonary bypass is very limited due to: (1) very few cortisol time point measurements over long periods (2) difficulties of sampling in low weight babies and (3) the concomitant use of glucocorticoids at anaesthesia induction. This lack of understanding is reflected in a lack of consensus on the utility of glucocorticoids perioperatively in cardiac surgery with the use of cardiopulmonary bypass.

**Methods:**

The Peacock Study is a prospective, two-centre, observational cohort study of 78 children (undergoing cardiopulmonary bypass procedures and non-surgical procedures - split by age/cyanosis) that aims to characterise in detail the hypothalamic-pituitary-adrenal axis physiology of children using the stress model of paediatric cardiac surgery. Also, we aim to correlate cortisol profiles with clinical outcome data. We herein describe the main study design and report the full cortisol profile of one child undergoing heart surgery, thus proving the feasibility of the method.

**Results:**

We used an automated, 24-h tissue microdialysis system to measure cortisol and cortisone, every 20 min. We herein report one cortisol profile of a child undergoing heart surgery. Besides, we measured serum cortisol and adrenocorticotrophic hormone at seven-time points for correlation. Tissue concentrations of cortisol increased markedly several hours after the end of surgery. We also noted an increase in the tissue cortisol/cortisone ratio during this response.

**Conclusion:**

We report for the first time, the use of an automated microdialysis sampling system to evaluate the paediatric adrenal response in children. Changes in cortisol and cortisone could be measured, and the concentration of cortisol in the tissues increased after the end of cardiac surgery. The method has wide application to measure other hormones dynamically and frequently without the limitation of the circulating blood volume. The data from the main study will clarify how these cortisol profiles vary with age, pathology, type of procedure and correlation to clinical outcomes.

**Trial registration:**

ISCRTN registry, number: 982586.

## Background

The primary neuroendocrine system that is activated during the stress response to surgery is the hypothalamic-pituitary-adrenal (HPA) axis [1]. Cortisol is the major stress hormone produced by the HPA axis to establish homeostasis during and after surgery. In the healthy individual, cortisol is released by the adrenal gland in a *circadian* manner but underlying this rhythm there is a *pulsatile* and *dynamic* cortisol release with a periodicity of approximately 1 h called an *ultradian* rhythm [2, 3]. The ultradian activation of the glucocorticoid receptor as opposed to the constant stimulation is necessary for optimal physiological cell regulation via a gene pulsing mechanism [4]. Pulsatile patterns of cortisol are altered in various conditions [5]. Previous work in patients undergoing adult heart surgery has demonstrated maintenance of serum cortisol pulsatility in the perioperative period [6]. Furthermore, animal research has shown a correlation between this plasma cortisol oscillation and the tissue, extracellular cortisol levels [7].

However, there is little research into the basic physiology of the HPA axis in children undergoing heart surgery. No study has examined ultradian rhythms of cortisol in children at all. Because of this knowledge gap, there is an ongoing debate around the modulation of the HPA axis function perioperatively to improve outcomes. Surgery with use of cardiopulmonary bypass (CPB) is a potent activator of the systemic inflammatory response and hence of the HPA axis. It is believed that this activation is augmented in the paediatric population [8]. Glucocorticoids have been used in paediatric heart surgery for more than 50 years, but there is still an ongoing debate around their utility [9]. The lack of knowledge of normal HPA axis responses in these patients is reflected in a lack of consensus around the utility of prophylactic glucocorticoid administration in paediatric heart surgery with cardiopulmonary bypass. In a national survey of practice, we found huge variability in corticosteroid administration, both between and within congenital heart surgery centres [10].

Our first difficulty in elucidating the role of steroids in paediatric cardiopulmonary bypass is our lack of understanding of the systemic inflammatory response to surgery and the difficulties of detecting a significant treatment effect in the context of a low mortality or morbidity results of contemporary paediatric heart surgery.

Another limitation is the lack of HPA axis physiology understanding we mentioned above. This knowledge gap stems from the difficulty of measuring plasma cortisol frequently enough due to the limitations of multiple blood samples taken from a low circulating volume - particularly in low weight babies. Indeed, an accurate assessment of the HPA axis requires frequent cortisol testing [11]. Most of the studies to date have measured cortisol at a few time points over long periods [8]. Another common limitation of these studies is that they attempted to assess HPA axis function after potent corticosteroids have been given preoperatively. Finally, defining abnormal HPA axis physiology by using limited points of cortisol or synthetic adreno-corticotropic-hormone stimulation tests is inaccurate in the context of the dynamic cortisol release. We herein describe our methods, study design, and provide the full cortisol profile of one child undergoing paediatric heart surgery.

## Methods/design

### Study aims

The Peacock Study aims to gain some understanding of the HPA axis physiology in children of various ages, not receiving perioperative steroids and undergoing various cardiac procedures (cardiac surgery or catheter procedures).

### The 24-h automated microdialysis system structure

**Fig. 1:**
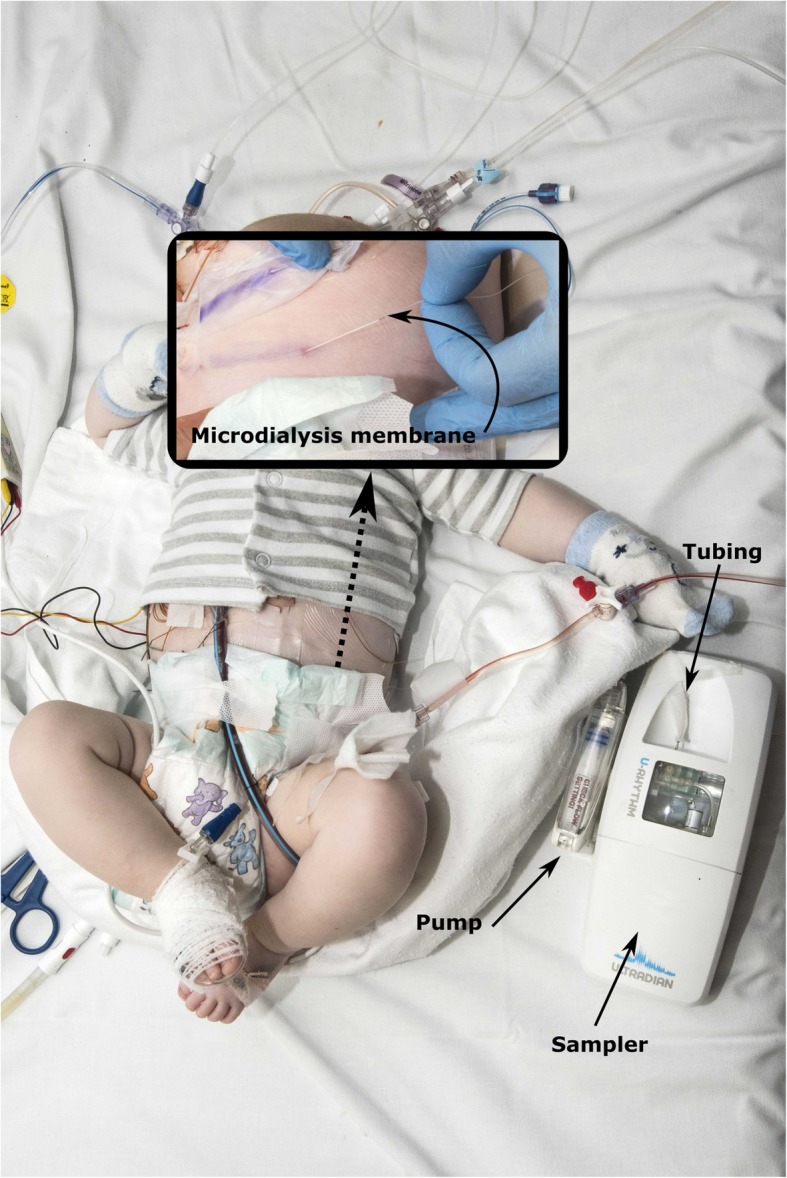
The microdialysis system in situ. Sampling in a 6-month-old baby that returned to the paediatric intensive care unit, after heart surgery

The ideal measurement of cortisol measurement in children must be acceptable for patients, parents, staff. Moreover, it has been reliable and automatic to facilitate a frequent cortisol measurement without affecting the circulating blood volume of the child. This is crucial, especially in neonates, that are in a critical state perioperatively and have blood taken as part of their routine care. Our research group has described a similar automated 24-h sampling system for the measurement of tissue free cortisol in healthy adult volunteers. The structure of this system has already been described in detail [12]. Briefly, the system is made from 3 parts (Fig. 1): (1) microdialysis pump (CMA 107 pump, MDialysis, Sweden); (2) microdialysis catheter (66 linear microdialysis catheter, MDialysis, Sweden), (3) sample collector/fractioner (Designworks, Windsor, UK) (4) (Fig. 1). These components are interconnected by fine tubing connectors that are assembled at the time insertion. The original design has undergone several modifications to allow successful application in the theatre environment where the catheter tubing is prone to kink or disconnect during surgery or patient transfer and as a result of clinical time pressures. Specifically, the connections were reinforced by silicone tube wrapping, and most of the components are preassembled to reduce the insertion time.

### How to the system works?

Using an aseptic technique, the microdialysis catheter is inserted in the left lower abdominal quadrant, in the subcutaneous tissue after induction of the anaesthesia. The microdialysis catheter is connected to a pump that infuses an isotonic dialysis fluid (T fluid, MDialysis, Sweden) at a pre-set rate (1 μl per minute). The free tissue cortisol/cortisone equilibrates across the microdialysis membrane into the perfusion fluid. At the other end, the fluid is collected by a fraction collector that essentially inserts a bubble of air in the column of fluid to separate the samples corresponding to specific time points.

### Tissue cortisol and cortisone analysis

After the end of the 24 h sampling period, the fluid samples (20 μl/per sample), separated by air bubbles, are collected inside of a 4,5 m tubing that is rolled onto a spool. This spool is opened, and careful interpretation of the timing and separation of each time point sample is undertaken. The samples are then pipetted in plates for further analysis.

Cortisol and cortisone were measured in the microdialysate by isotope dilution liquid chromatography-tandem mass spectrometry (LC-MS/MS) using cortisol-13C3 and cortisone-D7 as an internal standard. In short, 10 μL dialysate was pipetted into a 96-well plate (Waters), 50 μL of internal standard and 90 μL distilled water were added. The plate was mixed for ten minutes, and 2 μL was injected on the UPLC (Acquity) system equipped with a Kinetex Phenyl-Hexyl, 100 × 2.1 mm, 1.7 μm column (Phenomenex) in combination with a Xevo TQ-s with electrospray sionisation in selective reaction monitoring mode (Waters). For cortisol and cortisone, intra- and interassay variability were below 6 and 5%, respectively. Limit of quantification was 0.3 nmol/L for cortisol and 0.5 nmol/L for cortisone.

### Serum measurements

Serum cortisol, ACTH and CBG will be measured using competitive immunoassay methods. Cytokines will be measured using multiplex kits on a Luminex MAGPIX® system.

### Sample size calculation

The lack of previous studies does not allow power calculation on the expected variations in ultradian rhythmicity of the HPA axis in children. However, the use of multiple samples from the microdialysis provides great discriminatory power and the recent investigations from our group demonstrated very marked changes in pulse characteristics in a group of 20 adult patients undergoing coronary artery bypass grafting CABG compared to healthy individuals [6]. Furthermore, a study of 10 patients with obstructive sleep apnoea pre- and post- continuous positive airway pressure demonstrated significant changes in ACTH/cortisol ultradian patterns [13].

### The cardiac surgery cohort

This is a two-centre, descriptive study of the ultradian rhythms of cortisol that occur in neonates, infants and children. Forty-eight patients undergoing cardiac surgery will have 24-h tissue cortisol/cortisone microdialysis sampling after induction of anaesthesia up to 24 h. Also, serum cortisol, adrenocorticotrophic hormone (ACTH), interleukin 1 (IL-1), interleukin 4 (IL-4), interleukin 6 (IL-6), interleukin 8 (IL8), interleukin-10 (IL-10) and tumour necrosis factor-alpha (TNF-α) will be measured at 7-time points for infants and older children.

Several previous studies showed that inflammatory mediators have both direct and indirect effects on the HPA axis. Measurement of cytokines will allow us to assess this interaction and possibly allow us to build a mathematical model of how the HPA axis functions [6, 14].

The time points are pre-op assessment clinic blood, anaesthesia induction sample, pre-CPB, post-CPB, 6 h post anaesthesia induction, 12 h post anaesthesia induction and 24 h post anaesthesia induction. In neonates, due to circulating blood volume limits, we will measure the above variables at six timepoints only (the post-CPB sample is excluded). Cortisone binding globulin (CBG) will be measured at 4 time points only: pre-op assessment clinic blood, anaesthesia induction sample, 6 h post anaesthesia induction and 24 h post anaesthesia induction. The cohort will be further split into 4 groups. Neonates (6 cyanotic and 6 acyanotic heart disease), infants (6 cyanotic and 6 acyanotic heart disease), 6 children aged 1–5 and, 18 patients aged 10–16. For the 10–16 year’s age group, we will recruit only the patients operated in the morning. This group will be further split into three subgroups. The pre-pubertal group (6 children) and the postpubertal groups: 6 males and 6 females. Patients will be allocated to either pubertal or postpubertal group based on clinical criteria (e.g. presence of breast buds, periods, in girls and a testis volume greater than 4 mL in boys). Besides, the pre- and postpubertal groups (18 patients) will have one morning sample of follicle-stimulating hormone (FSH), lutein hormone (LH) and oestradiol or testosterone. Although pubertal status is assessed clinically by a trained paediatrician, measuring sex hormones gives us an objective measure to confirm their assessment.

### The cardiac investigation cohort

We will also recruit a cardiac investigation cohort of 30 children (no neonate group). This aims to assess if a less invasive procedure (e.g. no sternotomy and no cardiopulmonary bypass run) could influence the cortisol response in comparison to surgery. In this group, we will recruit 6 infants and 6 children (1–5-year-old) children. We will also recruit 18 patients in the age 10–16 years group. In this group, we will recruit 6 patients for the pre-pubertal group and 12 patients in the post-pubertal group (e.g. 6 males and 6 females).

Similarly, to the cardiac surgery cohort, all patients will undergo continuous tissue microdialysis sampling for cortisol and cortisone after anaesthesia induction up to 24 h, postoperatively. The cardiac investigation cohort patients will have only one basal pre-op sample via the cannula inserted for anaesthesia and microdialysis sampling for 24 h or until the patient is discharged. On this sample, we will measure serum cortisol, CBG, ACTH and the same interleukins measured in the surgical groups.

For both cardiac surgery and cardiac catheter cohorts, we decided to exclude the 5–10 years age range because there is not much surgery happening in this age range and we anticipated that it would be impossible to recruit these patients. This decision was based on an internal audit. For the same reason, we have excluded the neonate cardiac investigation cohort.

### Inclusion and exclusion criteria

The inclusion and exclusion criteria are outlined in Table 1.

**Table 1:** Inclusion and exclusion criteriaInclusion and exclusion criteria

Inclusion criteria	Exclusion criteria
Age 0–5 or 10–16 years (as per recruitment groups outlined above)	Current or recent (within three months) use of glucocorticoids
Use of CPB for the cardiac surgery cohort	Emergency operation
Weight above 2 kg	Disorders of the HPA axis
	Thyroid disease

### Outcomes

The primary outcome of the study is a description of how the cortisol/cortisone profiles are influenced by the age of the patient, the type of congenital heart defect and the type of procedure. The secondary outcomes include correlation of the cortisol/cortisone profiles with the various serum blood measurements and the clinical outcomes. We will record the following clinical outcomes: death, preoperative biventricular function, cardiac arrest, extracorporeal membrane oxygenation use, renal insufficiency (creatinine more than two times normal), hepatic insufficiency, duration of mechanical ventilation post-cardiac surgery, inotrope and vasopressors use, intensive care unit stay, hospital length of stay, infection, insulin use, fluid retention (daily weights).

### Consent

Consent +/− assent will be obtained according to patient age:

- 0–5 years: Parents/guardians of patients are expected to read and understand the parent/guardian information leaflet and (with the assistance of a member of the clinical team) explain the purposes and consequences of the study to the patient at a level suitable for their age, if appropriate. Parents/guardians are required to provide full, written, informed consent.
- 10–15 years: Patients who can read and understand will be provided with a patient information leaflet, and parents/guardians will be provided with a parent/guardian information leaflet. Parental consent must be obtained for enrolment to be valid. In parallel, where appropriate, the patients will be given a suitable form to provide written informed assent to participate in the study. Failure to complete and sign this assent form will be considered as a refusal to participate. This refusal should be respected.
- 16 years: Patients aged 16 years are legally responsible for providing their consent and will be provided with their information leaflet and consent form. Parental signed consent can be taken (also) using the parent/guardian consent form; however; this is not a necessity and alone is not enough for enrolment.

Patients can be formally enrolled, and consent/assent obtained at various points preoperatively.

### Ethics and dissemination

Ethical approval has been obtained from UK Research Ethics Committee, The South West – Frenchay Research Committee, REC reference: 11/H0107/9. The results will be published in peer-reviewed journals and presented at national and international conferences.

### Patient and public involvement

The research team involved parents of children admitted in the hospital in discussions about the acceptability of microdialysis catheter insertion perioperatively.

## Results

**Fig. 2:**
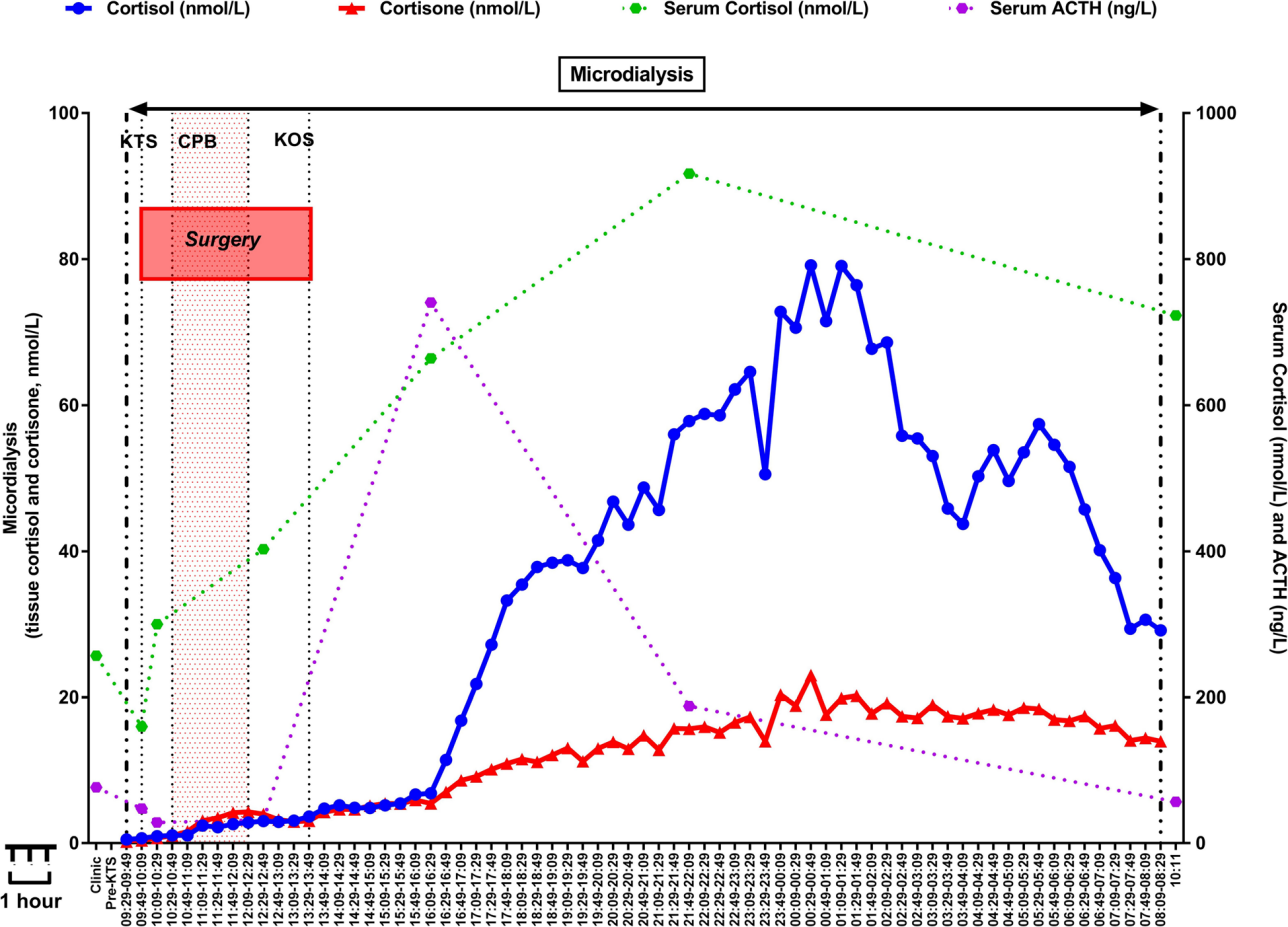
24-h cortisol profile of 2-year-old female undergoing ventricular septal defect. Abbreviations: KTS: knife to the skin, start of operation; CPB time is represented in a red shaded area. KOS – knife of skin time – end of the operation. In dotted green lines are illustrated the serum cortisol levels taken at the reference time points (seven). In the purple dotted line, the ACTH serum concentration taken at the reference time points (seven) is illustrated. On the right Y-axis, the serum cortisol/ACTH concentration is depicted. The blue, line denotes the tissue cortisol and the red line the tissue cortisone levels taken every 20 min using microdialysis

We report one cortisol profile of a child undergoing paediatric heart surgery using our automated microdialysis sampling system. We were able to measure cortisol and cortisone, every 20 min, for 24 h, using the tissue microdialysis system described above. We have noticed a marked increase in tissue cortisol concentrations several hours after the end of surgery. We have also noted an increase in the cortisol/cortisone ratios. Figure 2 depicts the cortisol profile of a 2-year-old female that underwent routine ventricular septal defect closure.

## Discussion

Frequent tissue cortisol and cortisone measurement, using an automated microdialysis system is feasible, as shown in the cortisol profile provided. This method enables us to obtain high-resolution cortisol and cortisone profiles at approximately 70 timepoints per 24 h. These preliminary results show that the method is feasible and safe, as shown in adult studies [12, 15, 16]. We will be assessing the tolerability and safety of the device in children as part of the main study.

In a recent literature review, we found that highest number of cortisol measurements achieved by all the studies published to date was 10-time points over 48 h and in almost all the studies glucocorticoids were administered on anaesthetic induction that certainly prevented a meaningful assessment [8]. The Peacock study aims to tackle these previous limitations by avoiding pre-operative steroids and frequently measuring cortisol/cortisone at the tissue level. Moreover, the use of this method does not affect the circulating volume and therefore allows sampling of patients with very low body weight, especially neonates. This is a physiologically distinct group that we know very little about. Hosein et al. [17] used a CMA 60 microdialysis catheter inserted in the anterior abdominal wall and connected to a microdialysis pump to measure tissue lactate and pyruvate, every 30 min in children 10 children (mean age 4.9 years) undergoing paediatric cardiac surgery followed by a manual collection of the samples. Our system enabled an automatic collection of the samples and first dynamic evaluation of the HPA axis function of children undergoing heart surgery.

In our cortisol profile (Fig. 2) we have noted three findings related to the cortisol release pattern: (1) several hours delay in the increase of the cortisol after the end of surgery (2) marked increase from basal of cortisol concentration at the tissue level and (3) an increase in the cortisol to cortisone ratio during this acute response.

The occurrence of tissue cortisol response after the surgery rather than during surgery is similar to a study that measured serum cortisol, every 10 min, in adults undergoing heart surgery with the use of cardiopulmonary bypass [6]. Therefore, we could further hypothesise that this delay in the HPA axis response is related to the inflammatory response rather than the effect of anaesthesia or surgery as it fits with the pattern of cytokine release during cardiopulmonary bypass [18]. In the current patient, we have not measured any cytokines; however, in the main study, we are collecting various cytokines at specific reference time points that will inform us more on this mechanism.

We have noted a marked increase from basal cortisol levels, approximately 80-fold from the basal level, and none of them has shown a suppression in the response. The magnitude of this cortisol response may suggest that the endogenous cortisol reserve of children is high enough to mount an adequate homeostatic response.

Finally, we have also noted increased cortisol to cortisone ratio. This could suggest an alteration of the activity of the 11β-Hydroxysteroid dehydrogenase (11ß-HSD) system. Several studies have demonstrated a similar increase in the cortisol to cortisone ratios in the days after cardiac surgery [19–21]. It is unclear if the 11ß-HSD isoenzyme type 2 that inactivates cortisol to cortisone is blocked or saturated by substrate overload or/and there is an activation of the 11ß-HSD isoenzyme type 1 that regenerates cortisol from cortisone. This hypothesis requires further investigation and will have to be validated on a larger number of patients that will be recruited and sampled using the methodswe herein describe.

Certainly, there is a dilutional effect of massive blood transfusion and fluid administration perioperatively that has a potential impact on tissue hormone levels. Furthermore, some patients will undergo modified ultrafiltration. However, this is a descriptive study of what children HPA axis function looks like during heart surgery. We will record and include this data in the final analysis.

## Conclusion

We have successfully obtained cortisol profiles of children undergoing paediatric heart surgery using an automated subcutaneous tissue microdialysis system. This novel method allowed us to obtain high-resolution cortisol profiles to get a better understanding of the HPA axis during acute stress. This preliminary data shows a marked increase in tissue cortisol levels several hours after surgery. Future data will also clarify how these cortisol profiles vary with age, pathology, type of procedure, inflammatory response and clinical outcomes.

## Data Availability

The datasets generate and analysed during the current feasibility study are not publicly available but are available from the corresponding author on reasonable request.

## Abbreviations

HPA: Hypothalamic-pituitary-adrenal axis
CPB: Cardiopulmonary bypass
LC-MS/MS: Isotope dilution liquid chromatography-tandem mass spectrometry
CABG: Coronary artery bypass grafting
ACTH: Adrenocorticotrophic hormone
IL-1: Interleukin 1
IL-4: Interleukin 4
IL-6: Interleukin 6
IL8: Interleukin 8
IL-10: Interleukin-10
TNF-α: Tumour necrosis factor alpha
CBG: Cortisone binding globulin
FSH: Follicle stimulating hormone
LH: Lutein hormone
11ß-HSD: 11β-Hydroxysteroid dehydrogenase
KTS: Knife to skin time
KOS: Knife off skin time
