# Correction to: The Peacock study: feasibility of the dynamic characterisation of the paediatric hypothalamic-pituitary-adrenal function during and after cardiac surgery

**DOI:** 10.1186/s12872-020-01561-7

**Published:** 2020-06-08

**Authors:** Daniel Paul Fudulu, Gianni Davide Angelini, Fani Fanoula Papadopoulou, Jonathan Evans, Terrie Walker-Smith, Ido Kema, Martijn Van Faassen, Serban Stoica, Massimo Caputo, Stafford Lightman, Benjamin Gibbison

**Affiliations:** 1 Department of Cardiac Surgery, Bristol Heart Institute, Bristol, UK; 2 Henry Welcome Laboratories for Integrative Neuroscience and Endocrinology, University of Bristol, Bristol, UK; 3 Department of Congenital Heart Surgery, Bristol Royal Hospital for Children, Bristol, UK; 4 Clinical Trial and Evaluation Unit, University of Bristol, Bristol, UK; 5 Department of Laboratory Medicine, University of Groningen, Groningen, Netherlands; 6 Department of Cardiac Anaesthesia, Bristol Heart Institute, Bristol, UK

**Correction to: BMC Cardiovasc Disord 20, 245 (2020)**

**https://doi.org/10.1186/s12872-020-01516-y**

Following publication of the original article [1], the authors identified an error in the author name of be Martijn van Faassen.

The incorrect author name is: Martijn van Fassen

The correct author name is: be Martijn van Faassen

The original article [1] has been updated.
